# Heart failure with improved versus persistently reduced left ventricular ejection fraction: A comparison of the BIOSTAT‐CHF (European) study with the ASIAN‐HF registry

**DOI:** 10.1002/ejhf.3378

**Published:** 2024-08-09

**Authors:** Thong Huy Cao, Wan Ting Tay, Donald J.L. Jones, John G.F. Cleland, Jasper Tromp, Johanna Elisabeth Emmens, Tiew‐Hwa Katherine Teng, Chanchal Chandramouli, Oliver Charles Slingsby, Stefan D. Anker, Kenneth Dickstein, Gerasimos Filippatos, Chim C. Lang, Marco Metra, Piotr Ponikowski, Nilesh J. Samani, Dirk J. Van Veldhuisen, Faiez Zannad, Inder S. Anand, Carolyn S.P. Lam, Adriaan A. Voors, Leong L. Ng

**Affiliations:** ^1^ Department of Cardiovascular Sciences, College of Life Sciences University of Leicester Leicester UK; ^2^ National Institute for Health and Care Research Leicester Biomedical Research Centre University Hospitals of Leicester NHS Trust, Glenfield Hospital Leicester UK; ^3^ Leicester van Geest Multi‐OMICS facility University of Leicester Leicester UK; ^4^ National Heart Centre Singapore and Duke–National University of Singapore Singapore Singapore; ^5^ Leicester Cancer Research Centre, University Hospitals of Leicester NHS Trust, Leicester Royal Infirmary University of Leicester Leicester UK; ^6^ British Heart Foundation Centre of Research Excellence, School of Cardiovascular and Metabolic Health, College of Medical, Veterinary and Life Sciences University of Glasgow Glasgow UK; ^7^ Saw Swee Hock School of Public Health National University of Singapore and the National University Health System Singapore Singapore; ^8^ Department of Cardiology University of Groningen Groningen The Netherlands; ^9^ Division of Cardiology and Metabolism, Department of Cardiology (CVK), and Berlin‐Brandenburg Center for Regenerative Therapies (BCRT), German Centre for Cardiovascular Research (DZHK) Partner Site Berlin Charité Universitätsmedizin Berlin Berlin Germany; ^10^ University of Bergen Stavanger University Hospital Stavanger Norway; ^11^ Department of Cardiology, Heart Failure Unit, Athens University Hospital Attikon, School of Medicine National and Kapodistrian University of Athens Athens Greece; ^12^ Division of Molecular and Clinical Medicine, Ninewells Hospital and Medical School University of Dundee Dundee UK; ^13^ Institute of Cardiology, Department of Medical and Surgical Specialties, Radiological Sciences and Public Health University of Brescia Brescia Italy; ^14^ Department of Heart Diseases Wroclaw Medical University and Cardiology Department, Military Hospital Wroclaw Poland; ^15^ Inserm CIC 1433 Université de Lorrain Nancy France; ^16^ Department of Medicine University of Minnesota Medical School and VA Medical Center Minneapolis MN USA

**Keywords:** Clinical outcome, Heart failure, Heart failure with improved ejection fraction, Left ventricular ejection fraction, Heart failure with reduced ejection fraction, Predictive model, Predictor

## Abstract

**Aims:**

We investigated the prevalence, clinical characteristics, and prognosis of patients with heart failure (HF) with improved ejection fraction (HFimpEF).

**Methods and results:**

We used data from BIOSTAT‐CHF including patients with a left ventricular ejection fraction (LVEF) ≤40% at baseline who had LVEF re‐assessed at 9 months. HFimpEF was defined as a LVEF >40% and a LVEF ≥10% increase from baseline at 9 months. We validated findings in the ASIAN‐HF registry. The primary outcome was a composite of time to HF rehospitalization or all‐cause mortality. In BIOSTAT‐CHF, about 20% of patients developed HFimpEF, that was associated with a lower primary event rate of all‐cause mortality (hazard ratio [HR] 0.52, 95% confidence interval [CI] 0.28–0.97, *p* = 0.040) and the composite endpoint (HR 0.46, 95% CI 0.30–0.70, *p* < 0.001) compared with patients who remained in persistent HF with reduced ejection fraction (HFrEF). The findings were similar in the ASIAN‐HF (HR 0.40, 95% CI 0.18–0.89, *p* = 0.024, and HR 0.29, 95% CI 0.17–0.48, *p* < 0.001). Five independently common predictors for HFimpEF in both BIOSTAT‐CHF and ASIAN‐HF were female sex, absence of ischaemic heart disease, higher LVEF, smaller left ventricular end‐diastolic and end‐systolic diameter at baseline. A predictive model combining only five predictors (absence of ischaemic heart disease and left bundle branch block, smaller left ventricular end‐systolic and left atrial diameter, and higher platelet count) for HFimpEF in the BIOSTAT‐CHF achieved an area under the curve of 0.772 and 0.688 in the ASIAN‐HF (due to missing left atrial diameter and platelet count).

**Conclusions:**

Approximately 20–30% of patients with HFrEF improved to HFimpEF within 1 year with better clinical outcomes. In addition, the predictive model with clinical predictors could more accurately predict HFimpEF in patients with HFrEF.

## Graphical abstract

Findings of LVEF improvement, clinical outcomes, predictors and a predictive model comparing HFimpEF and persistent HFrEF in BIOSTAT‐CHF and validated in the ASIAN‐HF registry. Approximately 20‐30% of patients with HFrEF improved within 1 year (HFimpEF) with better clinical outcomes. In addition, the predictive model with clinical predictors (absence of IHD and LBBB, smaller LVESD and LA diameter, and higher platelet count) could more accurately predict HFimpEF in patients with HFrEF and be easily applied in clinical practice. Common predictors in red. AUC, area under the curve; GT, glutamyl transferase; HF, heart failure; HFimpEF, heart failure with improved ejection fraction; HFrEF, heart failure with reduced ejection fraction; HR, hazard ratio; IHD: ischemic heart disease; LA, left atrium; LBBB, left bundle branch block; LVEDD, left ventricular end‐diastolic diameter; LVEF, left ventricular ejection fraction; LVESD, left ventricular end‐systolic diameter.

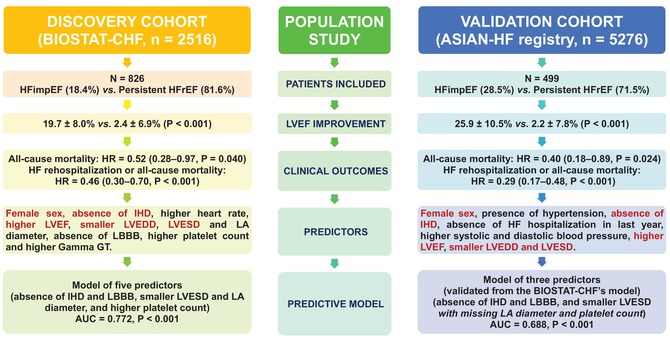

## Introduction

Heart failure (HF) therapy can improve left ventricular ejection fraction (LVEF) in patients with HF with reduced ejection fraction (HFrEF) that is associated with better outcomes.^1^, ^2^, ^3^, ^4^, ^5^, ^6^ According to a recent consensus report of the International Societies of HF, the experts recommended defining patients with improved LVEF as a separate subcategory because the improvement of LVEF has led to an interest in the long‐term outcomes and management of these patients as compared to those whose LVEF did not improve with treatment.^7^ Here, HF with improved ejection fraction (HFimpEF) was defined as HF with a baseline LVEF of ≤40%, a ≥10‐point increase from baseline in LVEF and a second measurement of LVEF of >40%.^7^ Patients with HFimpEF may be distinct in terms of clinical demographics, biochemical laboratory, and outcomes from those with HFrEF or HF with preserved LVEF.^2^, ^3^, ^8^ However, data on HFimpEF have been sparse. Early identification of patients with HFimpEF and persistent HFrEF during diagnosis would be useful in clinical decision‐making and therapeutic management. Therefore, our aims in this study were to investigate the clinical characteristics, predictors and the prognostic impact of improved LVEF on outcomes. Additionally, as secondary aims, we determined predictive models and recommended a LVEF threshold for improvement of LVEF in patients with HFimpEF as compared to those with persistent HFrEF. To do this, we analysed clinical data from a multinational European, prospective clinical study (BIOSTAT‐CHF)^9^ and validated our findings in an independent multinational cohort (ASIAN‐HF registry).^10^, ^11^ The findings could inform the optimization of pharmacotherapy and improve outcomes in patients with HF.

## Methods

### Study design and participants

The EU FP7 funded BIOSTAT‐CHF (A systems BIOlogy Study to TAilored Treatment in Chronic Heart Failure) project was a multinational, prospective clinical study in Europe.^9^ The main aim of this project was to employ a systems biology approach (including demographics, biomarkers, proteomics and genetics) in order to identify poor outcomes in HF patients with standard therapy described elsewhere.^12^, ^13^, ^14^, ^15^, ^16^, ^17^, ^18^ This study was conducted according to the Declaration of Helsinki and was approved by local and national ethics committees. All patients (*n* = 2516) in this study provided written informed consent. Patients from 69 centres in 11 European countries who met inclusion and exclusion criteria according to the European Society of Cardiology (ESC) guidelines were recruited into this study.^19^ In brief, patients more than 18 years old with presentations of HF symptoms and a LVEF ≤40% and/or B‐type natriuretic peptide (BNP) >400 pg/ml or N‐terminal pro‐B‐type natriuretic peptide (NT‐proBNP) >2000 pg/ml who were receiving suboptimal HF treatment (untreated or treated with ≤50% of the target dose of both angiotensin‐converting enzyme inhibitors [ACEi] or angiotensin receptor blockers [ARB] and beta‐blockers) were invited into the study (online supplementary *Figure* *S1*). The patients were optimized with standard treatment for HF by initiating or up‐titrating ACEi or ARB and beta‐blockers during the first 3 months. Clinical endpoints such as HF rehospitalization and mortality were assessed.^9^ The assessment for LVEF was performed at the beginning and at 9 months of the study. Patients with HFimpEF were those with a baseline LVEF ≤40%, a ≥10‐point increase from baseline in LVEF at a second measurement of LVEF >40% at 9 months.^7^ A flow diagram of selected patients in this study is displayed in online supplementary *Figure* *S2*, and online supplementary *Table* *S1* presents the baseline characteristics of HF patients included or excluded in BIOSTAT‐CHF.

### Outcomes

Clinical endpoints were assessed for patients with HF in BIOSTAT‐CHF.^9^ The primary endpoint included a composite of time to HF rehospitalization or all‐cause mortality. After the 9‐month visit, a standard clinic follow‐up or contact via telephone was performed every 6 months. The median follow‐up time for the primary endpoint was 21 (interquartile range [IQR] 15–27) months.

### Validation analysis

The findings of this study were validated in the ASIAN‐HF registry that was a prospective multinational study of 5276 patients of ≥18 years old with symptomatic HF (stage C) from 11 Asian regions (44 centres).^10^, ^11^ This analysis included 499 patients with LVEF ≤40% at the baseline visit and available LVEF measurement at 1‐year follow‐up. ASIAN‐HF also complied with the Declaration of Helsinki and was approved by medical ethics committees of participating centres. All patients provided written informed consent. Online supplementary *Table* *S2* presents the baseline characteristics of HF patients included and excluded in ASIAN‐HF.

### Statistical analysis

The statistical software SPSS 26.0 for Windows was used for statistical analyses in this study. Continuous variables were displayed using mean ± standard deviation (SD) for normally distributed continuous variables, median (IQR) for non‐normally distributed variables, and categorical variables were expressed as numbers with percentages. Values were compared using Student's *t*‐tests, Mann–Whitney U tests or Chi‐square tests for group comparisons as appropriate. Cox proportional hazard models were employed to calculate hazard ratios (HR), adjusted for the effect of potential confounders. The predictive models for HFimpEF were built using logistic regression with backward and forward stepwise selection of the clinical variables. All statistical tests were performed two‐tailed, and a *p*‐value <0.05 was considered statistically significant.

### Role of the funding source

The study's funders had no role in study design, data collection, data analysis, data interpretation, or writing of the report. The corresponding authors had full access to all the data in the study and had final responsibility for the decision to submit for publication.

## Results

### Changes in left ventricular ejection fraction

**Figure 1:**
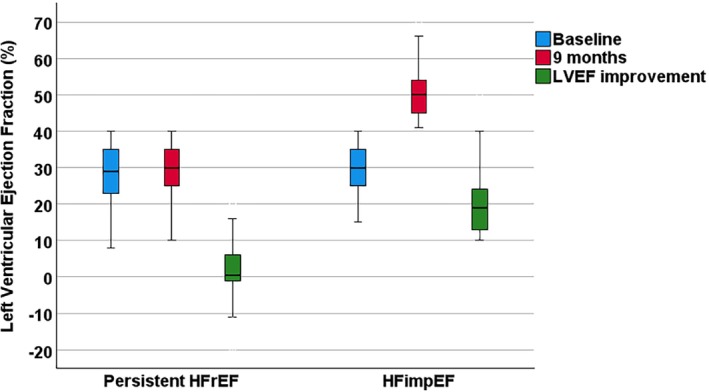
Left ventricular ejection fraction (LVEF) at baseline and at 9 months and improvement in comparisons between heart failure with improved ejection fraction (HFimpEF) and persistent heart failure with reduced ejection fraction (HFrEF) in BIOSTAT‐CHF.

Of 2516 patients enrolled in BIOSTAT‐CHF, 2243 (89.2%) had an echocardiographic assessment of LVEF at baseline; 2008 of those met the inclusion criteria of a LVEF ≤40%. A total of 329 patients died before the visit at 9 months for a reassessment of LVEF, and 871 patients had a LVEF assessment at 9 months, of whom 152 (18.4%) patients were classified as HFimpEF according to the universal definition and classification of HF^7^ and 674 patients (81.6%) had persistent HFrEF (online supplementary *Figure* *S2*). By definition, there was a highly significant LVEF improvement of 19.7 ± 8.0% in patients with HFimpEF (29.8 ± 6.8% at baseline vs. 49.5 ± 5.7% at 9 months) as compared to patients with persistent HFrEF (Δ 2.4 ± 6.9%, 27.8 ± 7.2% at baseline vs. 30.2 ± 7.2% at 9 months) (*p* < 0.001) (*Table* *1* and *Figure* *1*).

**Table 1:** Patient characteristics at baseline and 9 months in comparison between heart failure with improved ejection fraction and persistent heart failure with reduced ejection fraction groups in BIOSTAT‐CHF

Characteristics	Baseline			9 months		
	HFimpEF (*n* = 152)	Persistent HFrEF (*n* = 674)	*p*‐value	HFimpEF (*n* = 152)	Persistent HFrEF (*p* = 674)	*p*‐value
Age, years	63.7 ± 12.8	66.3 ± 11.6	**0.017**			
Male sex, *n* (%)	105 (69.1)	542 (80.4)	**0.002**			
Medical history, *n* (%)						
Hypertension	80 (52.6)	421 (62.5)	**0.025**			
Ischaemic heart disease	38 (25.0)	312 (46.3)	**<0.001**			
Valvular surgery	11 (7.2)	46 (6.8)	0.856			
Atrial fibrillation	65 (42.8)	238 (35.3)	0.085			
Diabetes mellitus	31 (20.4)	201 (29.8)	**0.019**			
Stroke	9 (5.9)	55 (8.2)	0.351			
Peripheral arterial disease	5 (3.3)	61 (9.1)	**0.018**			
COPD	22 (14.5)	103 (15.3)	0.802			
HF hospitalization in last year	41 (27.0)	210 (31.2)	0.311			
Clinical profile						
Heart rate, bpm	85.9 ± 23.8	77.5 ± 17.2	**<0.001**	70.5 ± 14.4	72.0 ± 15.7	0.291
Systolic blood pressure, mmHg	125.8 ± 21.6	124.3 ± 20.2	0.411	130.8 ± 21.8	122.2 ± 19.8	**<0.001**
Diastolic blood pressure, mmHg	78.0 ± 14.2	76.5 ± 11.9	0.208	77.2 ± 12.1	74.9 ± 11.6	**0.027**
BMI, kg/m^2^	27.3 ± 5.4	27.8 ± 4.9	0.310	27.8 ± 5.4	28.1 ± 5.6	0.569
NYHA class III/IV, *n* (%)	68 (45.0)	349 (52.5)	0.098	14 (9.7)	174 (26.3)	**<0.001**
Orthopnoea, *n* (%)	41 (27.0)	150 (22.3)	0.216	3 (2.3)	50 (8.0)	**0.018**
Pulmonary rales, *n* (%)	64 (43.8)	293 (44.3)	0.926	6 (5.2)	73 (12.1)	**0.028**
Peripheral oedema, *n* (%)	60 (49.6)	269 (49.9)	0.949	10 (9.4)	128 (25.1)	**<0.001**
Jugular venous pressure, *n* (%)	26 (25.0)	115 (24.8)	0.972	0 (0.0)	46 (9.9)	**0.002**
Third heart sound, *n* (%)	11 (7.3)	69 (10.3)	0.261	1 (0.8)	31 (5.0)	**0.030**
Hepatomegaly, *n* (%)	18 (11.8)	106 (15.8)	0.223	1 (0.8)	50 (8.1)	**0.003**
Echocardiography						
LVEF, %	29.8 ± 6.8	27.8 ± 7.2	**0.003**	49.5 ± 5.7	30.2 ± 7.2	**<0.001**
LVEDD, mm	59.3 ± 8.0	64.3 ± 8.2	**<0.001**	54.3 ± 6.8	64.0 ± 8.7	**<0.001**
LVESD, mm	48.3 ± 8.2	52.7 ± 9.7	**<0.001**	39.3 ± 7.1	51.8 ± 10.1	**<0.001**
LA diameter, mm	43.8 ± 8.6	47.7 ± 7.6	**<0.001**	41.6 ± 6.0	47.9 ± 7.7	**<0.001**
ECG, *n* (%)						
LBBB	18 (12.0)	163 (24.4)	**<0.001**	14 (9.4)	135 (21.2)	**<0.001**
RBBB	14 (9.3)	42 (6.3)	0.184	9 (6.0)	45 (7.1)	0.656
LVH	22 (14.7)	81 (12.1)	0.400	13 (8.7)	60 (9.4)	0.793
Laboratory						
Serum creatinine, μmol/L	89.2 (79.0–107.0)	100.0 (82.0–126.0)	**<0.001**	88.6 (76.7–114.8)	106.0 (86.6–132.6)	**<0.001**
Blood urea, mmol/L	8.9 (6.6–14.5)	11.3 (7.5–18.0)	**<0.001**	8.1 (6.0–12.1)	11.1 (7.3–17.9)	**<0.001**
eGFR, ml/min^−1^	72.6 ± 23.5	68.7 ± 26.5	0.092	71.8 ± 26.1	63.7 ± 24.6	**0.001**
Sodium, mEq/L	139.5 ± 3.3	139.7 ± 3.9	0.528	139.8 ± 3.0	139.5 ± 3.7	0.441
Potassium, mEq/L	4.2 ± 0.5	4.3 ± 0.6	**0.044**	4.4 ± 0.4	4.5 ± 0.6	**0.008**
Glucose, mg/dl	6.7 ± 2.7	6.8 ± 2.7	0.870	6.7 ± 2.7	6.6 ± 2.4	0.784
HbA1c, %	6.6 ± 1.4	6.5 ± 1.5	0.737	6.5 ± 1.3	6.3 ± 1.5	0.488
Total cholesterol, mmol/L	4.6 ± 1.3	4.4 ± 1.3	0.191	4.8 ± 1.2	4.6 ± 1.4	0.291
HDL cholesterol, mmol/L	1.1 ± 0.4	1.1 ± 0.4	0.898	1.2 ± 0.3	1.1 ± 0.4	0.096
LDL cholesterol, mmol/L	2.8 ± 1.0	2.7 ± 1.0	0.433	2.8 ± 1.0	2.8 ± 1.3	0.900
Triglycerides, mmol/L	1.5 ± 1.0	1.5 ± 0.9	0.873	1.8 ± 1.2	1.9 ± 1.5	0.684
Haemoglobin, g/dl	13.7 ± 1.9	13.5 ± 1.7	0.296	13.4 ± 1.6	13.3 ± 1.7	0.507
Red blood cell count, million/mm^3^	4.5 ± 0.6	4.5 ± 0.7	0.965	4.4 ± 0.5	4.5 ± 0.6	0.405
White blood cell count, 1000/mm^3^	8.2 ± 3.0	8.1 ± 2.7	0.849	7.2 ± 2.3	7.7 ± 2.5	0.069
Platelet count, 1000/mm^3^	249.8 ± 87.8	221.6 ± 81.1	**0.001**	242.9 ± 77.9	220.6 ± 66.5	**0.010**
Total bilirubin, μmol/L	12.3 (8.3–18.2)	13.0 (9.5–18.8)	0.265	10.2 (7.2–13.0)	12.0 (8.7–17.0)	**0.005**
AST, U/L	28.0 (21.0–37.0)	23.0 (18.0–32.0)	**<0.001**	24.0 (20.0–29.5)	22.0 (18.0–28.0)	0.107
ALT, U/L	33.0 (21.0–51.0)	24.5 (16.3–35)	**<0.001**	24.0 (18.7–29.5)	21.0 (16.0–30.0)	**0.045**
Gamma GT, U/L	61.5 (30.3–128.8)	41.0 (24.8–81.3)	**0.002**	38.0 (22.0–72.3)	35.0 (23.5–66.0)	0.494
Alkaline phosphatase, μg/L	77.5 (63.4–109.3)	81.0 (61.0–117.0)	0.900	79.5 (68.0–114.5)	99.0 (68.0–141.6)	**0.041**
TSH, mU/L	1.7 (1.1–2.6)	1.9 (1.0–3.0)	0.581	1.7 (1.1–2.3)	1.7 (1.0–3.0)	0.358
FT4, pmol/L	15.5 ± 4.8	15.7 ± 7.5	0.909	15.5 ± 2.7	14.7 ± 4.0	0.353
Troponin I, μg/L	0.03 (0.01–0.08)	0.03 (0.01–0.10)	0.613	0.01 (0.002–0.014)	0.02 (0.01–0.03)	**0.013**
Troponin T, μg/L	0.03 (0.02–0.04)	0.03 (0.01–0.06)	0.884	0.014 (0.012–0.025)	0.026 (0.013–0.066)	0.140
NT‐proBNP, pg/ml	3874.0 (2147.5–6646.0)	3412.5 (1622.3–7811.8)	0.587	791.0 (173.3–1985.5)	1810.0 (668.4–5251.0)	**<0.001**
Medication, *n* (%)						
ACEi/ARB	120 (78.9)	536 (79.5)	0.874	142 (93.4)	627 (93.0)	0.862
Beta‐blocker	127 (83.6)	578 (85.8)	0.488	142 (93.4)	644 (95.5)	0.270
MRA	73 (48.0)	391 (58.0)	**0.025**	67 (44.1)	437 (65.1)	**<0.001**
Loop diuretics	152 (100)	671 (99.6)	0.410	120 (78.9)	635 (94.6)	**<0.001**
Digoxin	30 (19.7)	111 (16.5)	0.333	23 (15.1)	116 (17.3)	0.522

Values are mean ± standard deviation, or median (interquartile range), unless otherwise specified.

ACEi, angiotensin‐converting enzyme inhibitor; ALT, alanine transaminase; ARB, angiotensin receptor blocker; AST, aspartate transaminase; BMI, body mass index; COPD, chronic obstructive pulmonary disease; ECG, electrocardiography; eGFR, estimated glomerular filtration rate; FT4, free thyroxine; GT, glutamyl transferase; HbA1c, glycated haemoglobin; HDL, high‐density lipoprotein; HF, heart failure; HFimpEF, heart failure with improved ejection fraction; HFrEF, heart failure with reduced ejection fraction; LA, left atrial; LBBB, left bundle branch block; LDL, low‐density lipoprotein; LVEDD, left ventricular end‐diastolic diameter; LVEF, left ventricular ejection fraction; LVESD, left ventricular end‐systolic diameter; LVH, left ventricular hypertrophy; MRA, mineralocorticoid receptor antagonist; NT‐proBNP, N‐terminal pro‐B‐type natriuretic peptide; NYHA, New York Heart Association; RBBB, right bundle branch block; TSH, thyroid‐stimulating hormone.

### Demographic and clinical characteristics

**Figure 2:**
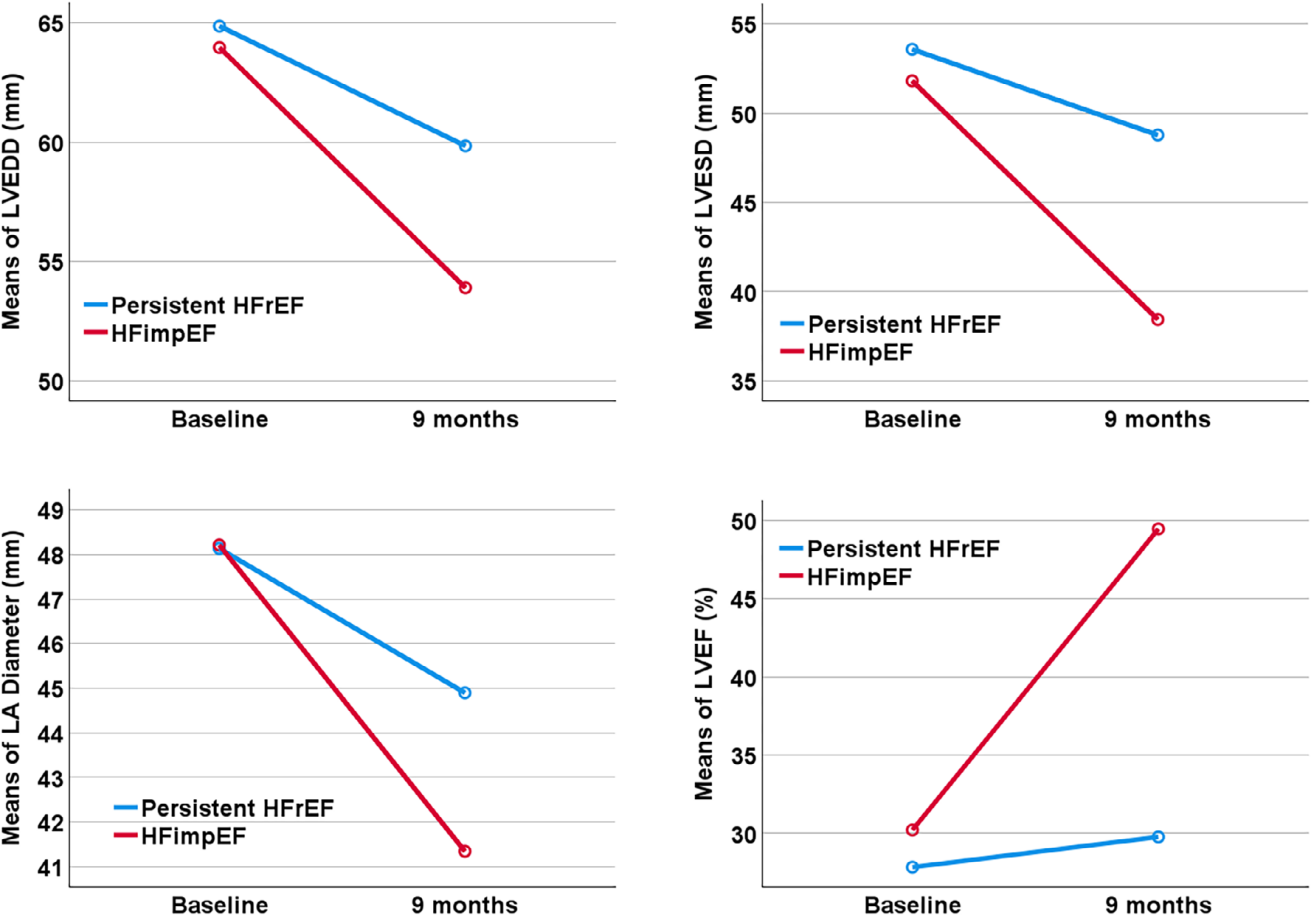
Left ventricular end‐diastolic diameter (LVEDD), left ventricular end‐systolic diameter (LVESD), left atrial (LA) diameter and left ventricular ejection fraction (LVEF) in comparisons between baseline and 9 months according to heart failure with improved ejection fraction (HFimpEF) and persistent heart failure with reduced ejection fraction (HFrEF) in BIOSTAT‐CHF.

The baseline demographic and clinical characteristics of patients with HFimpEF and persistent HFrEF are described in *Table* *1*. As compared to patients with persistent HFrEF, patients with HFimpEF were more likely to be younger (*p* = 0.017), female (*p* = 0.002), but less likely to have ischaemic heart disease (IHD) (*p* < 0.001), hypertension (*p* = 0.025), diabetes mellitus (*p* = 0.019) and peripheral arterial disease (*p* = 0.018). Heart rate was higher in patients with HFimpEF (*p* < 0.001). Patients with HFimpEF had a higher LVEF (29.8 ± 6.8% vs. 27.8 ± 7.2%, *p* = 0.003) and a smaller left ventricular end‐diastolic diameter (LVEDD) (*p* < 0.001), left ventricular end‐systolic diameter (LVESD) (*p* < 0.001) and left atrial (LA) diameter (*p* < 0.001) as compared to patients with persistent HFrEF (*Figure* *2*). The HFimpEF group (vs. persistent HFrEF group) had a lower prevalence of left bundle branch block (LBBB) on electrocardiography (*p* < 0.001). In clinical laboratory variables, there were significant differences for blood urea (*p* < 0.001), serum creatinine (*p* < 0.001), serum potassium (*p* = 0.044) and higher platelet count (*p* = 0.001), aspartate transaminase (*p* < 0.001), alanine transaminase (*p* < 0.001) and gamma glutamyl transferase (GT) (*p* = 0.002) in patients with HFimpEF as compared to those with persistent HFrEF. Fewer patients with HFimpEF were treated with mineralocorticoid receptor antagonist (MRAs) as compared to the persistent HFrEF group at baseline (*p* = 0.025).

Clinical characteristics of patients with HFimpEF and persistent HFrEF at 9 months are described and compared with baseline clinical characteristics in *Table* *1*. All clinical and chemical laboratory variables that had significant differences between both HF groups at baseline remained significant at 9 months, except for differences in heart rate (*p* = 0.291), aspartate transaminase (*p* = 0.107) and gamma GT (*p* = 0.494). At 9 months, the HFimpEF group had a better symptomatic profile for orthopnoea (*p* = 0.018), pulmonary rales (*p* = 0.028), peripheral oedema (*p* < 0.001), jugular venous pressure (*p* = 0.002), third heart sound (*p* = 0.030), hepatomegaly (*p* = 0.003) and a lower prevalence of New York Heart Association (NYHA) class III or IV (*p* < 0.001). In addition, the HFimpEF group had better clinical chemistry profiles of estimated glomerular filtration rate (*p* = 0.001), total bilirubin (*p* = 0.005), alkaline phosphatase (*p* = 0.041), troponin I (*p* = 0.013) and NT‐proBNP (pg/ml) (791.0 [173.3–1985.5] vs. 1810.0 [668.4–5251.0], *p* < 0.001). Furthermore, there was a significant decrease of LVEDD, LVESD, and LA diameter (mm) of 5.5 ± 7.0, 9.5 ± 8.4 and 2.5 ± 8.3 in patients with HFimpEF as compared to those with persistent HFrEF (Δ 0.8 ± 5.2, 1.5 ± 7.1 and −0.4 ± 6.7 at *p* < 0.001, *p* < 0.001 and *p* = 0.003, respectively) (*Table* *1* and *Figure* *2*).

### Predictors and predictive models for improvement of left ventricular ejection fraction in BIOSTAT‐CHF

Independent predictors for development to HFimpEF are shown in *Table* *2*. The predictors were female sex, absence of IHD, higher heart rate, higher LVEF, smaller LVEDD, LVESD and LA diameter, absence of LBBB, higher platelet count, higher gamma GT and lower rate of MRA use. We developed a model with these 10 predictors (except for lower rate of MRA use that might be a confounding variable) using logistic regression in which all these predictors were entered simultaneously. The predictive capability of this model for HFimpEF had an area under the curve (AUC) value of 0.810 (95% confidence interval [CI] 0.739–0.881, *p* < 0.001) (online supplementary *Figure* *S3A*). Furthermore, from a logistic regression (backward and forward selection) analysis, we identified a predictive model for HFimpEF that included only five predictors: absence of IHD and LBBB, smaller LVESD and LA diameter, and higher platelet count. The performance of this model for the predictive probability of HFimpEF in HFrEF patients achieved an AUC of 0.772 (95% CI 0.715–0.829, *p* < 0.001) (online supplementary *Figure* *S3B*). The Hosmer and Lemeshow test was performed using binary logistic regression that showed predictions made by the model fit well with observed group memberships (Chi‐square *p*‐value = 0.246). The predictive model classified 82.0% of cases overall in accuracy.

**Table 2:** Predictors of heart failure with improved ejection fraction in patients with heart failure with reduced ejection fraction at baseline in BIOSTAT‐CHF

Predictors	OR	95% CI	*p*‐value*
Female sex	1.842	1.220–2.782	0.004
Ischaemic heart disease	0.461	0.306–0.695	<0.001
Heart rate	1.017	1.008–1.026	<0.001
LVEF	1.049	1.021–1.078	<0.001
LVEDD	0.916	0.891–0.943	<0.001
LVESD	0.939	0.912–0.966	<0.001
LA diameter	0.941	0.915–0.967	<0.001
LBBB	0.431	0.251–0.739	0.002
Platelet count	1.003	1.000–1.005	0.018
Gamma GT	1.003	1.000–1.005	0.032
MRA	0.664	0.458–0.962	0.030

CI, confidence interval; GT, glutamyl transferase; LA, left atrial; LBBB, left bundle branch block; LVEDD, left ventricular end‐diastolic diameter; LVEF, left ventricular ejection fraction; LVESD, left ventricular end‐systolic diameter; MRA, mineralocorticoid receptor antagonist; OR, odds ratio.

* Multivariable model: adjusted for age, sex, ischaemic heart disease, systolic blood pressure, diabetes mellitus and estimated glomerular filtration rate.

### Clinical outcomes

**Figure 3:**
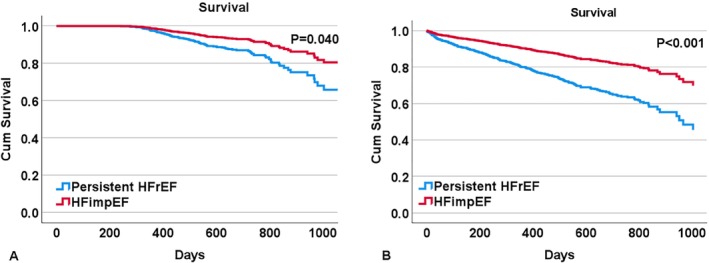
Clinical outcomes according to heart failure with improved ejection fraction (HFimpEF) and persistent heart failure with reduced ejection fraction (HFrEF) in BIOSTAT‐CHF. (*A*) Cox regression survival curves for all‐cause mortality according to HFimpEF and persistent HFrEF (792 patients [95.9%] were eligible for the Cox regression model). (*B*) Cox regression survival curves for the composite of heart failure rehospitalization or all‐cause mortality according to HFimpEF and persistent HFrEF (781 patients [94.6%] were eligible for the Cox regression model).

Over a median follow‐up of 21 (IQR 15–27) months, patients with HFimpEF had better prognosis during the follow‐up time for the primary endpoints. Crude deaths occurred in 11 (7.2%) patients with HFimpEF compared to 97 (14.4%) in the persistent HFrEF group (*p* = 0.018). *Figure* *3* shows Cox regression analysis for all‐cause mortality (HR 0.52, 95% CI 0.28–0.97, *p* = 0.040) and the composite of HF rehospitalization or all‐cause mortality (HR 0.46, 95% CI 0.30–0.70, *p* < 0.001) for HFimpEF versus persistent HFrEF. Furthermore, the cardiovascular and non‐cardiovascular mortality rates were lower in patients with HFimpEF as compared to those with persistent HFrEF (4.6% vs. 10.4% and 2.6% vs. 4.0%, *p* < 0.001) (online supplementary *Table* *S3*).

### Validation of findings in the ASIAN‐HF registry

To confirm the findings in BIOSTAT‐CHF, we employed the ASIAN‐HF registry. Of 499 patients in the ASIAN‐HF registry, 28.5% of patients developed HFimpEF and 71.5% had persistent HFrEF. There was also a highly significant LVEF improvement of 25.9 ± 10.5% in patients with HFimpEF at 1 year as compared to those with persistent HFrEF (Δ 2.2 ± 7.8%, *p* < 0.001). Baseline demographic and clinical characteristics of patients with HFimpEF and persistent HFrEF in the ASIAN‐HF cohort (online supplementary *Table* *S4*) were similar to BIOSTAT‐CHF. Independent predictors of the development of HFimpEF in ASIAN‐HF are reported in online supplementary *Table* *S5*. Patients with HFimpEF in ASIAN‐HF also had better prognosis for all‐cause mortality (4.9% vs. 13.5%; HR 0.40, 95% CI 0.18–0.89, *p* = 0.024) and the composite endpoint of HF rehospitalization or all‐cause mortality (12.0% vs. 38.4%; HR 0.29, 95% CI 0.17–0.48, *p* < 0.001) as compared to those with persistent HFrEF (460 patients [92.2%] were eligible for the Cox regression model). Furthermore, the cardiovascular and non‐cardiovascular mortality rates in ASIAN‐HF were lower in the HFimpEF group as compared to the persistent HFrEF group (4.9% vs. 10.9% and 0% vs. 2.5%, *p* = 0.037 and *p* = 0.056, respectively) (online supplementary *Table* *S3*). These confirmed the results in BIOSTAT‐CHF (*Figure* *3*). In addition, we tested the predictive model for HFimpEF in the ASIAN‐HF cohort that was developed in the BIOSTAT‐CHF cohort including five predictors: absence of IHD and LBBB, smaller LVESD and LA diameter, and higher platelet count. Unfortunately, LA diameter and platelet count were not available in the ASIAN‐HF cohort. Therefore, this predictive model with missing LA diameter and platelet count only yielded an AUC of 0.688 (95% CI 0.634–0.742, *p* < 0.001) (online supplementary *Figure* *S3C*). Moreover, the predictive model using absence of IHD and LBBB, and smaller LVESD was able to classify 72.9% of cases overall in accuracy.

### Predictive models for improvement of left ventricular ejection fraction in BIOSTAT‐CHF and validated in the ASIAN‐HF registry

We found six common predictors in BIOSTAT‐CHF and the ASIAN‐HF registry for HFimpEF prediction in patients with HFrEF, including female sex, absence of IHD, higher LVEF, smaller LVEDD and LVESD, and lower rate of MRA use at baseline. A predictive model for HFimpEF was developed by combining five of these common predictors (except for lower rate of MRA use) using logistic regression analysis. The performance of this model for the predictive probability of HFimpEF in patients with HFrEF achieved an AUC of 0.702 (95% CI 0.648–0.757, *p* < 0.001) in BIOSTAT‐CHF (online supplementary *Figure* *S3D*) that was also validated in ASIAN‐HF with an AUC of 0.682 (95% CI 0.629–0.736, *p* < 0.001) (online supplementary *Figure* *S3E*).

### Comparisons of clinical outcomes between a ≥5% threshold and a ≥10% change from baseline left ventricular ejection fraction

Sensitivity analyses were performed to compare clinical outcomes between a ≥5 and 10‐point increase from baseline LVEF. We found that, when using the 5% LVEF threshold for HFimpEF definition in the BIOSAT‐CHF cohort, patients were also associated with a lower primary event rate of all‐cause mortality (6.5% vs. 14.4%; HR 0.46, 95% CI 0.25–0.84, *p* = 0.011) and the composite endpoint of HF rehospitalization or all‐cause mortality (14.5% vs. 33.1%; HR 0.41, 95% CI 0.28–0.61, *p* < 0.001) as compared to those with persistent HFrEF (online supplementary *Figure* *S4*). These results were similar to the clinical outcome results using the 10% LVEF threshold for HFimpEF classification. In addition, using the 5% LVEF threshold for HFimpEF classification, patients with HFimpEF in ASIAN‐HF also had better prognosis for all‐cause mortality (5.5% vs. 13.5%; HR 0.43, 95% CI 0.21–0.88, *p* = 0.020) and the composite endpoint of HF rehospitalization or all‐cause mortality (12.7% vs. 38.4%; HR 0.30, 95% CI 0.19–0.48, *p* < 0.001) as compared to those with persistent HFrEF, and these results confirmed the findings in BIOSTAT‐CHF.

## Discussion

This study investigated the demographic and clinical characteristics, predictors and predictive models, and clinical outcomes in patients with HFimpEF in comparison with persistent HFrEF. Approximately 20% of patients with HFrEF in BIOSTAT‐CHF developed HFimpEF within 9 months as compared to 30% at 1 year in the ASIAN‐HF registry. More patients in ASIAN‐HF had a LVEF improvement as compared to BIOSTAT‐CHF, possibly due to the longer follow‐up time before LVEF re‐assessment (*Graphical Abstract*). In a recent study presented at the Heart Failure Society of America 2021 Annual Scientific Meeting on a cohort of 131 patients with HFrEF in the Middle East‐Gulf Region, Alsindi *et al*.^20^ reported that 27.5% of HFrEF patients became HFimpEF after a follow‐up of 12 months. These authors also found that patients with HFimpEF developed a significantly higher LVEF at 12‐month follow‐up (48.6 ± 7.2% vs. 28.7 ± 8.7%, *p* < 0.001). In a systematic review including 9491 HF patients from nine studies by He *et al*.,^5^ 22.6% of patients with HFrEF became HFimpEF during an average follow‐up of 3.8 years, indicating a prevalence similar to BIOSTAT‐CHF. Liu and colleagues also reported a prevalence of LVEF improvement of 37.2% in patients with HFrEF during a median follow‐up of 17 months.^6^

### Predictors and predictive models for heart failure with improved ejection fraction

In this study, we found five common predictors (excluding lower rate of MRA use that might be a confounding variable) that were the same in BIOSTAT‐CHF and ASIAN‐HF registry for prediction of a LVEF improvement (HFimpEF) in patients with HFrEF at baseline. The predictors were female sex, absence of IHD, higher LVEF, smaller LVEDD and LVESD. Previous reports also showed similar results regarding these predictors for LVEF improvement in patients with HFrEF.^1^, ^2^, ^3^, ^6^, ^21^ In addition, Alsindi *et al*.^20^ reported that patients with HFimpEF were less likely to have IHD. Kong and colleagues also found that patients with HFimpEF had a higher prevalence of female sex in a cohort of 230 HF patients.^4^ These predictors of HFimpEF would provide important information that could be used for LVEF improvement prediction, risk stratification and treatment guidance in patients with HFrEF in order to customize therapy, thus maximizing the treatment benefits in patients with HF. Moreover, patients with HFimpEF in our study also showed a reverse in cardiac remodelling, whereas patients with persistent HFrEF did not. The evidence for this was demonstrated by a significant reduction in LVEDD, LVESD and LA diameter. LA reverse remodelling may occur across all stages of progression of HF after initiation of medical therapy and be associated with better clinical outcomes, as reported in a review by Inciardi *et al*.^22^ These authors suggested that the left atrium should be a target of treatment or rather a surrogate measurement of the severity of cardiac dysfunction.

Heart failure is a syndrome that involves many pathophysiological processes. In addition, due to the heterogeneity of clinical populations (age, sex, ethnicity and comorbidity), it is unlikely that a single clinical variable could identify patients with HFimpEF from those with HFrEF. Therefore, we developed a model combining only five clinical variables (absence of IHD and LBBB, smaller LVESD and LA diameter, and higher platelet count) in the BIOSTAT‐CHF study that provided better prediction of HFimpEF for patients with HFrEF as compared to each single clinical variable with an AUC of 0.772. However, the parameters of LA diameter and platelet count were not available in the ASIAN‐HF registry. Therefore, the validation of this predictive model with missing LA diameter and platelet count only yielded an AUC of 0.688 in the ASIAN‐HF cohort. The absence of IHD in the predictive model could be a very strong predictor for HFimpEF that was also reported in previous studies.^2^, ^3^, ^6^, ^20^, ^21^ The LVESD and LA diameter in the predictive model suggest that it is difficult to reverse once remodelling occurs. This predictive model would be easily applied to clinical practice to predict patients with HFrEF who will improve LVEF to customize therapy to individuals.

### Prognosis in patients with heart failure with improved ejection fraction

Patients with HFimpEF had a better prognosis than those with persistent HFrEF in this study, as evidenced by a highly reduced risk of all‐cause mortality and the composite endpoint of HF rehospitalization or all‐cause mortality. In addition. both cardiovascular and non‐cardiovascular mortality rates were lower in patients with HFimpEF as compared to persistent HFrEF. These results were similar in both the BIOSTAT‐CHF study and the ASIAN‐HF registry. Our findings are consistent with previous reports that demonstrated a superior long‐term clinical prognosis in patients with HFimpEF.^1^, ^2^, ^3^ Patients with HFimpEF had a lower mortality rate (116 deaths, 16.1%) as compared with those with persistent HFrEF (270 deaths, 34.2%), HF with mildly reduced ejection fraction (214 deaths, 33.5%), and HF with preserved ejection fraction (149 deaths, 31.6%) during 4‐year follow‐up in Park et al.'s study.^3^ Florea and colleagues also reported that 397 (11.3%) patients died during a median (IQR) of 2 (1.6–2.4) years and the mortality rate ratio was 0.49 (0.31–0.76, *p* = 0.002) for patients with HFimpEF.^2^ Patients with HFimpEF showed lower rate of HF hospitalization or all‐cause mortality as a primary endpoint as compared to those with persistent HFrEF in a study of 230 patients by Kong *et al*.^4^ (6.2% vs. 22.4%; HR 0.24, 95% CI 0.13–0.46, *p* < 0.001). Moreover, He and colleagues reported a lower risk of mortality (HR 0.44, 95% CI 0.33–0.60), and the composite endpoint of hospitalization and mortality (HR 0.56, 95% CI 0.44–0.73) in patients with HFimpEF as compared to those with HFrEF.^5^ In a study by Liu *et al*.,^6^ patients with HFimpEF showed lower all‐cause mortality (22.1% vs. 31.1%, *p* = 0.019; HR 0.67, 95% CI 0.47–0.97) as compared to patients without HFimpEF. In a recent study (a pre‐specified analysis of the DELIVER trial), Vardeny *et al*.^23^ reported that patients with HFimpEF (previous LVEF ≤40%) had similar event rates to those with a LVEF consistently >40% (prior LVEF >40%) in terms of primary composite endpoint (cardiovascular mortality or worsening HF, HR 0.99, 95% CI 0.85–1.15), cardiovascular mortality (HR 0.94, 95% CI 0.75–1.19), HF hospitalization (HR 1.01, 95% CI 0.84–1.21), all‐cause mortality (HR 1.03, 95% CI 0.87–1.20) and the composite of cardiovascular mortality and recurrent HF events (rate ratio 0.98, 95% CI 0.82–1.17). However, in their study HFimpEF was defined as patients whose LVEF improved from ≤40% to >40% and compared to patients with a LVEF consistently >40% (prior LVEF >40%, not persistent HFrEF).

### Was a ≥5‐point increase as good as the 10% change from baseline left ventricular ejection fraction?

One of our findings in this study was that using the 5% LVEF improvement threshold for HFimpEF definition in both BIOSTAT‐CHF and ASIAN‐HF was as good as using the ≥10% change from baseline LVEF in terms of a lower primary event rate of all‐cause mortality and the composite endpoint of HF rehospitalization or all‐cause mortality. However, the 5% LVEF threshold is the margin of measurement error using echocardiography that may be a limitation to its utility.

### Future perspectives

Five common predictors (female sex, absence of IHD, higher LVEF, smaller LVEDD and LVESD) could be used for prediction of a LVEF improvement (HFimpEF) in patients with HFrEF. The predictive model combining absence of IHD and LBBB, smaller LVESD and LA diameter, and higher platelet count in this study could predict HFimpEF in patients with HFrEF with more accuracy and would be easily applied to clinical practice for prediction of LVEF improvement in patients with HFrEF. Instead of the traditional one‐size‐fits‐all treatment recommendation, early identification and intervention of HFimpEF and persistent HFrEF would provide useful information to allow clinicians to customize therapy to individuals that will not only require less frequent visits for patients at low risk (HFimpEF) but also allow management efforts to be focused on high‐risk patients (persistent HFrEF), thus maximizing the treatment benefits in patients with HF.

### Strengths and limitations

This study has several limitations. First, some confounding factors might not have been assessed because this study is a post hoc analysis of a prospective cohort study. Second, there may be survivor bias because only patients with HF who were still alive and had an echocardiographic assessment of LVEF at 9 months were selected for the study. Therefore, many HF patients with poor outcomes who died before 9 months were already excluded from the study. Third, only Caucasian patients were included in the BIOSTAT‐CHF study design. In addition, the ASIAN‐HF registry was a prospective multinational study from 11 Asian regions^10^, ^11^ that was employed for validation of BIOSTAT‐CHF findings. There are differences in genetic background, body mass index, pharmacokinetics and drug response between Caucasian and Asian populations. Therefore, this was also a limitation for validating the findings from the BIOSTAT‐CHF cohort. Moreover, the number of patients in the ASIAN‐HF registry that met inclusion criteria for comparison with BIOSTAT‐CHF was smaller (*n* = 499) as well as missing several clinical variables such as LA diameter and platelet count, so these may limit the validity of complex predictive models including multiple variables. Fourth, the BIOSTAT‐CHF project and ASIAN‐HF registry were conducted between 2010–2015 and 2012–2015, respectively, so standard therapies were based on the ESC guidelines of 2008 that recommended the use of MRAs only for patients who had a NYHA class IV in the past 6 months (level B evidence) and no specific up‐titration strategy for MRAs. In addition, new drugs such as sodium–glucose cotransporter 2 inhibitors were not prescribed for HF patients at that particular time.^19^ Therefore, the findings in this study should be interpreted in that scenario.

This study also has specific strengths. The BIOSTAT‐CHF project is a well‐designed, multinational, prospective clinical study in which patients had echocardiography assessments at baseline and 9‐month follow‐up. Echocardiographic assessment of LVEF at 9 months may be the appropriate time for the detection of HFimpEF after initiation or up‐titration of medications.^24^

## Conclusions

To our knowledge, this was the first large international study using the universal definition and classification of HF for HFimpEF by Bozkurt *et al*.^7^ HFimpEF is a distinct HF phenotype with better clinical outcomes than persistent HFrEF. The predictive model with clinical predictors (absence of IHD and LBBB, smaller LVESD and LA diameter, and higher platelet count) could more accurately predict HFimpEF in patients with HFrEF. Early identification of HFimpEF and persistent HFrEF in this study would allow customization of therapy to individuals, instead of the traditional one‐size‐fits‐all treatment recommendation.

### Funding

This work was funded by the European Commission (FP7‐242209‐BIOSTAT‐CHF; EudraCT 2010‐020808‐29) and supported by the John and Lucille van Geest Foundation, and the National Institute for Health and Care Research Leicester Biomedical Research Centre.

**Conflict of interest**: T.H.C. was funded by the National Institute for Health and Care Research Leicester Biomedical Research Centre. J.G.F.C. has received committee fees and/or research grants from Abbott, Amgen, AstraZeneca, Bayer, Bristol Myers Squibb, GlaxoSmithKline, Medtronic, Myokardia, Novartis International AG, Philips, Pharmacosmos, PharmaNord, Sanofi, Servier, Stealth Biopharmaceuticals, Torrent Pharmaceuticals and Vifor. S.D.A. reports grants from Vifor and Abbott Vascular, and fees for consultancy from Vifor, Bayer, Boehringer Ingelheim, Impulse Dynamics, Novartis, Respicardia and Servier. G.F. has received committee fees and/or research grants from Novartis, Bayer, Vifor, and Servier. C.C.L. has received consultancy fees and/or research grants from Amgen, AstraZeneca, Merck Sharp & Dohme, Novartis, and Servier. M.M. has received consulting honoraria from Bayer, Novartis, and Servier. D.J.V.V. has received board membership fees or travel expenses from Novartis and Johnson & Johnson. A.A.V. received consultancy fees and/or research grants from Amgen, AstraZeneca, Bayer, Boehringer Ingelheim, Cytokinetics, GlaxoSmithKline, Myokardia, Novartis, Roche Diagnotics and Servier. L.L.N. has received grants from the John and Lucille Van Geest Foundation and National Institute for Health and Care Research. All other authors have nothing to disclose.

## Supporting information


**Data S1.** Supporting information.
