# Supplementary material for: Heart failure with improved versus persistently reduced left ventricular ejection fraction: A comparison of the BIOSTAT‐CHF (European) study with the ASIAN‐HF registry
Source: Eur J Heart Fail. 2024 Aug 9;26(12):2518–28. doi: 10.1002/ejhf.3378 (PMC11683861; doi:10.1002/ejhf.3378)
Supplement: Supplementary file 1 — Data S1. Supporting information. [file EJHF-26-2518-s001.docx]

Supplementary Material

**Heart failure with improved versus persistently reduced left ventricular ejection fraction: a comparison of the BIOSTAT-CHF (European) and ASIAN-HF Registry**

**Authors**

Thong Huy Cao, MD, MSc, PhD^1,2,3^, Wan Ting Tay, MAppStat^4^, Donald J. L. Jones, PhD^1,3,5^, John G. F. Cleland, MD, PhD^6^, Jasper Tromp, MD, PhD^4,7^, Johanna Elisabeth Emmens, MD, PhD^8^, Tiew-Hwa Katherine Teng, PhD^4^, Chanchal Chandramouli, PhD^4^, Oliver Charles Slingsby, BSc^1,2,3^, Stefan D. Anker, MD, PhD^9^, Kenneth Dickstein, MD, PhD^10^, Gerasimos Filippatos, MD, PhD^11^, Chim C. Lang MD^12^, Marco Metra, MD^13^, Piotr Ponikowski, MD, PhD^14^, Nilesh J. Samani, MD^1,2^, Dirk J. Van Veldhuisen, MD, PhD^8^, Faiez Zannad, MD^15^, Inder S Anand, MD, D Phil^16^, Carolyn S. P. Lam, MD, PhD^4^ on behalf of the ASIAN-HF investigators, Adriaan A. Voors, MD, PhD^8^, Leong L. Ng, MD^1,2,3^

Supplementary Table S1: Baseline characteristics of patients with HF that were included and excluded in BIOSTAT-CHF.

| Characteristics | Baseline | | |
| --- | --- | --- | --- |
|  | **Included**  **(n = 826)** | **Excluded**  **(n = 1690)** | **P value** |
| Age (years) | 65.8 ± 11.9 | 70.5 ± 11.7 | **<0.001** |
| Male sex, n (%) | 647 (78.3) | 1199 (70.9) | **<0.001** |
| Medical history | | | |
| Hypertension, n (%) | 501 (60.7) | 1068 (63.2) | 0.217 |
| Ischemic heart disease, n (%) | 350 (42.4) | 781 (46.2) | 0.069 |
| Valvular surgery, n (%) | 57 (6.9) | 122 (7.2) | 0.771 |
| Atrial fibrillation, n (%) | 303 (36.7) | 840 (49.7) | **<0.001** |
| Diabetes mellitus, n (%) | 232 (28.1) | 587 (34.7) | **<0.001** |
| Stroke, n (%) | 64 (7.7) | 169 (10.0) | 0.067 |
| Peripheral arterial disease, n (%) | 66 (8.0) | 207 (12.2) | **0.001** |
| COPD, n (%) | 125 (15.1) | 311 (18.4) | **0.042** |
| HF hospitalisation in last year, n (%) | 251 (30.4) | 543 (32.1) | 0.377 |
| Clinical profile | | | |
| Heart rate (bpm) | 79.0 ± 18.9 | 80.4 ± 19.8 | 0.047 |
| Systolic blood pressure (mm Hg) | 124.5 ± 20.5 | 124.8 ± 22.6 | 0.385 |
| Diastolic blood pressure (mm Hg) | 76.8 ± 12.3 | 74.0 ± 13.8 | **<0.001** |
| BMI (kg/m^2^) | 27.7 ± 5.0 | 28.0 ± 5.7 | 0.082 |
| NYHA class III/IV, n (%) | 417 (51.1) | 1105 (67.8) | **<0.001** |
| Orthopnoea, n (%) | 191 (23.2) | 688 (40.8) | **<0.001** |
| Pulmonary rales, n (%) | 357 (44.2) | 934 (57.1) | **<0.001** |
| Peripheral oedema, n (%) | 329 (49.8) | 927 (64.4) | **<0.001** |
| Jugular venous pressure, n (%) | 141 (24.9) | 413 (38.0) | **<0.001** |
| Third heart sound, n (%) | 80 (9.7) | 167 (9.9) | 0.895 |
| Hepatomegaly, n (%) | 124 (15.0) | 234 (13.9) | 0.445 |
| Echocardiography | | | |
| LVEF (%) | 28.2 ± 7.1 | 32.7 ± 11.9 | **<0.001** |
| LVED diameter (mm) | 63.4 ± 8.4 | 59.8 ± 10.3 | **<0.001** |
| LVES diameter (mm) | 51.9 ± 9.6 | 48.7 ± 12.3 | **<0.001** |
| Left atrial diameter (mm) | 47.0 ± 7.9 | 47.6 ± 8.1 | 0.077 |
| ECG | | | |
| LBBB (%) | 181 (22.2) | 373 (22.3) | 0.919 |
| RBBB (%) | 56 (6.9) | 154 (9.2) | 0.046 |
| LVH (%) | 103 (12.6) | 250 (15.0) | 0.109 |
| Laboratory | | | |
| Serum creatinine (µmol/L) | 97.2 (80.2-23.0) | 105.0 (86.0-133.0) | **<0.001** |
| Blood urea (mmol/L) | 10.8 (7.4-16.8) | 11.7 (7.7-18.9) | **0.007** |
| eGFR (ml/min^-1^) | 139.7 ± 3.8 | 138.9 ± 4.1 | **<0.001** |
| Sodium (mEq/L) | 4.3 ± 0.5 | 4.3 ± 0.6 | **<0.001** |
| Potassium (mEq/L) | 6.8 ± 2.7 | 7.4 ± 3.2 | 0.180 |
| Glucose (mg/dL) | 6.5 ± 1.5 | 6.7 ± 1.4 | **<0.001** |
| HbA1c (%) | 4.5 ± 1.3 | 4.1 ± 1.3 | 0.180 |
| Total cholesterol (mmol/L) | 1.1 ± 0.4 | 1.1 ± 0.4 | **<0.001** |
| LDL cholesterol (mmol/L) | 2.7 ± 1.0 | 2.5 ± 1.1 | **0.002** |
| HDL cholesterol (mmol/L) | 1.5 ± 1.0 | 1.4 ± 1.1 | 0.074 |
| Triglycerides | 13.5 ± 1.8 | 13 ± 1.9 | 0.112 |
| Hemoglobin (g/dL) | 4.5 ± 0.7 | 4.4 ± 0.7 | **<0.001** |
| Red blood cell count (million per mm^3^) | 8.1 ± 2.8 | 8.4 ± 3.0 | **<0.001** |
| White blood cell count (1000 per mm^3^) | 227.2 ± 83.2 | 226.3 ± 92.3 | **0.039** |
| Platelet count (1000 per mm^3^) | 139.7 ± 3.8 | 138.9 ± 4.1 | 0.826 |
| Total bilirubin (µmol/L) | 12.9 (9.2-18.6) | 15.0 (10.1-23.0) | **<0.001** |
| ASAT (U/L) | 24.0 (18.0-34.0) | 26.0 (20.0-36.0) | **<0.001** |
| ALAT (U/L) | 25.0 (17.6-39.0) | 25.0 (16.0-37.0) | 0.227 |
| Gamma GT (U/L) | 44.0 (26.0-84.0) | 63.0 (30.0-123.0) | **<0.001** |
| Alkaline phosphatase (µg/L) | 80.0 (62.0-116.0) | 87.0 (68.8-119.0) | **0.011** |
| TSH (mU/L) | 1.8 (1.1-2.9) | 1.9 (1.2-3.1) | 0.159 |
| FT4 (pmol/L) | 15.6 ± 6.9 | 17.8 ± 7.9 | **<0.001** |
| Troponin I (µg/L) | 0.03 (0.01-0.10) | 0.04 (0.02-0.11) | **0.009** |
| Troponin T (µg/L) | 0.03 (0.02-0.05) | 0.03 (0.02-0.06) | 0.110 |
| NT-proBNP (pg/mL) | 3561 (1771.3-7328.8) | 4448 (2547.8-8845.3) | **<0.001** |
| Medication | | | |
| ACEi/ARB, n (%) | 656 (79.4) | 1164 (68.9) | **<0.001** |
| Beta-blocker, n (%) | 705 (85.4) | 1388 (82.1) | **0.042** |
| MRA, n (%) | 464 (56.2) | 875 (51.8) | **0.038** |
| Loop diuretics, n (%) | 823 (99.6) | 1681 (99.5) | 0.563 |
| Digoxin, n (%) | 141 (17.1) | 350 (20.7) | 0.031 |

ACEi, angiotensin converting enzyme inhibitor; ARB, angiotensin receptor blocker; AST, aspartate transaminase; ALT, alanine transaminase; BMI, body mass index; BNP, brain natriuretic peptide; COPD, chronic obstructive pulmonary disease; ECG: Electrocardiography: eGFR, estimated glomerular filtration rate; FT4, free thyroxine; HbA1c, glycated hemoglobin; HDL, high density lipoprotein; HFimpEF, heart failure with improved ejection fraction; HFrEF, heart failure with reduced ejection fraction; IHD: ischemic heart disease; LBBB, left bundle branch block; LDL, low density lipoprotein; LVED, left ventricular end diastolic; LVEF, left ventricular ejection fraction; LVES, left ventricular end systolic; MRA, mineralocorticoid receptor antagonist; NT-proBNP, NT-pro brain natriuretic peptide; NYHA, New York Heart Association; RBBB, right bundle branch block; TSH, thyroid stimulating hormone; LVH, left ventricular hypertrophy.

Values are means ± standard deviations (SD), medians (IQR, interquartile range [25^th^ to 75^th^ percentile range]) or numbers (percentages).

Supplementary Table S2: Baseline characteristics of patients with HF that were included and excluded in ASIAN-HF.

| Characteristics | Baseline | | |
| --- | --- | --- | --- |
|  | **Included**  **(n =499)** | **Excluded**  **(n =3803)** | **P value** |
| Age (years) | 62.1 ± 13.6 | 60.3 ± 13.0 | **0.003** |
| Male sex, n (%) | 373 (74.7) | 2941 (77.3) | 0.200 |
| Medical history | | | |
| Hypertension, n (%) | 245 (49.1) | 1953 (51.4) | 0.340 |
| Ischemic heart disease, n (%) | 192 (38.5) | 1973 (51.9) | **<0.001** |
| Atrial fibrillation, n (%) | 149 (29.9) | 609 (16.0) | **<0.001** |
| Diabetes mellitus, n (%) | 174 (34.9) | 1559 (41.0) | **0.009** |
| Stroke, n (%) | 42 (8.4) | 225 (5.9) | **0.030** |
| Peripheral arterial disease, n (%) | 13 (2.6) | 106 (2.8) | 0.820 |
| COPD, n (%) | 51 (10.2) | 283 (7.4) | **0.029** |
| HF hospitalisation in last year, n (%) | 334 (66.9) | 2231 (58.7) | **0.002** |
| Clinical profile | | | |
| BMI (kg/m^2^) | 23.9 ± 4.7 | 25.1 ± 5.5 | **<0.001** |
| NYHA class III/IV, n (%) | 129 (29.5) | 1098 (32.0) | 0.270 |
| Heart rate (bpm) | 77.8 ± 15.7 | 80.2 ± 16.3 | **0.002** |
| Systolic blood pressure (mm Hg) | 115.3 ± 19.3 | 119.0 ± 20.3 | **<0.001** |
| Diastolic blood pressure (mm Hg) | 68.9 ± 12.1 | 73.0 ± 12.7 | **<0.001** |
| Orthopnoea, n (%) | 73 (14.8) | 783 (20.8) | **0.002** |
| Pulmonary rales, n (%) | 47 (9.4) | 610 (16.0) | **<0.001** |
| Peripheral oedema, n (%) | 102 (20.4) | 863 (22.7) | 0.260 |
| Jugular venous pressure, n (%) | 54 (10.8) | 588 (15.5) | **0.006** |
| Third heart sound, n (%) | 56 (11.2) | 394 (10.4) | 0.550 |
| Hepatomegaly, n (%) | 30 (6.0) | 193 (5.1) | 0.370 |
| Echocardiography | | | |
| LVEF (%) | 27.3 ± 7.1 | 27.3 ± 7.1 | 0.900 |
| LVED diameter (mm) | 62.3 ± 9.7 | 60.8 ± 10.1 | **0.003** |
| LVES diameter (mm) | 53.3 ± 10.6 | 51.7 ± 11.1 | **0.006** |
| Left atrial volume (ml) | 97.0 ± 67.4 | 64.8 ± 38.4 | **<0.001** |
| ECG | | | |
| LBBB (%) | 47 (9.7) | 520 (14.5) | **0.004** |
| RBBB (%) | 31 (6.4) | 335 (9.4) | **0.033** |
| LVH (%) | 97 (20.1) | 918 (25.6) | **0.009** |
| Laboratory | | | |
| eGFR (ml/min^-1^) | 66.0 (46.1-82.8) | 62.4 (43.6-82.7) | 0.210 |
| Sodium (mEq/L) | 139.0 (137.0-141.0) | 138 (136.0-141.0) | **<0.001** |
| Potassium (mEq/L) | 4.3 (3.9-4.6) | 4.2 (3.9-4.6) | 0.200 |
| Hemoglobin (g/dL) | 13.3 (11.7-14.6) | 13.0 (11.5-14.4) | **0.009** |
| Medication | | | |
| ACEi/ARB, n (%) | 410 (82.2) | 2860 (76.2) | **0.003** |
| Beta-blocker, n (%) | 446 (89.4) | 2839 (75.7) | **<0.001** |
| MRA, n (%) | 313 (62.7) | 2170 (57.8) | **0.037** |
| Loop diuretics, n (%) | 375 (77.8) | 2913 (80.7) | 0.130 |
| Digoxin, n (%) | 103 (21.4) | 941 (26.1) | **0.026** |

ACEi, angiotensin converting enzyme inhibitor; ARB, angiotensin receptor blocker; BMI, body mass index; COPD, chronic obstructive pulmonary disease; ECG: Electrocardiography: eGFR, estimated glomerular filtration rate; HFimpEF, heart failure with improved ejection fraction; HFrEF, heart failure with reduced ejection fraction; LBBB, left bundle branch block; LVED, left ventricular end diastolic; LVEF, left ventricular ejection fraction; LVES, left ventricular end systolic; MRA, mineralocorticoid receptor antagonist; New York Heart Association; RBBB, right bundle branch block; LVH, left ventricular hypertrophy.

Values are means ± standard deviations (SD), medians (IQR, interquartile range [25^th^ to 75^th^ percentile range]) or numbers (percentages).

**Supplementary Table S3:** **Cardiovascular and non-cardiovascular mortality rates in patients with HFimpEF and persistent HFrEF in BIOSTAT-CHF and ASIAN-HF.**

| **Event** | **HFimpEF** | **HFrEF** | **P value** |
| --- | --- | --- | --- |
| **BIOSTAT-CHF** | | | |
| **CV death** | 7 (4.6%) | 70 (10.4%) | **<0.001** |
| **Non-CV death** | 4 (2.6%) | 27 (4.0%) | **<0.001** |
| **ASIAN-HF** | | | |
| **CV death** | 7 (4.9%) | 39 (10.9%) | **0.037** |
| **Non-CV death** | 0 (0%) | 9 (2.5%) | 0.056 |

Supplementary Table S4: Baseline characteristics of patients with HF in comparisons between HFimpEF and persistent HFrEF in ASIAN-HF.

| Characteristics | Baseline | | |
| --- | --- | --- | --- |
|  | **HFimpEF**  **(n =142)** | **Persistent**  **HFrEF**  **(n =357)** | **P value** |
| Age (years) | 60.5 (15.0) | 62.8 (13.0) | 0.091 |
| Male sex, n (%) | 96 (67.6) | 277 (77.6) | **0.021** |
| Medical history | | | |
| Hypertension, n (%) | 85 (59.9) | 160 (44.8) | **0.002** |
| Ischemic heart disease, n (%) | 38 (26.8) | 154 (43.1) | **<0.001** |
| Atrial fibrillation, n (%) | 35 (24.6) | 114 (31.9) | 0.110 |
| Diabetes mellitus, n (%) | 41 (28.9) | 133 (37.3) | 0.076 |
| Stroke, n (%) | 9 (6.3) | 33 (9.2) | 0.290 |
| Peripheral arterial disease, n (%) | 5 (3.5) | 8 (2.2) | 0.420 |
| COPD, n (%) | 17 (12.0) | 34 (9.5) | 0.420 |
| HF hospitalisation in last year, n (%) | 73 (51.4) | 261 (73.1) | **<0.001** |
| Clinical profile | | | |
| BMI (kg/m^2^) | 24.1 (4.8) | 23.8 (4.7) | 0.580 |
| NYHA class III/IV, n (%) | 31 (24.0) | 98 (31.7) | 0.110 |
| Heart rate (bpm) | 79.7 (16.3) | 77.1 (15.4) | 0.089 |
| Systolic blood pressure (mm Hg) | 119.6 (20.0) | 113.6 (18.7) | **0.002** |
| Diastolic blood pressure (mm Hg) | 71.4 (12.8) | 67.9 (11.7) | **0.004** |
| Orthopnoea, n (%) | 16 (11.3) | 57 (16.2) | 0.170 |
| Pulmonary rales, n (%) | 9 (6.3) | 38 (10.6) | 0.140 |
| Peripheral oedema, n (%) | 26 (18.3) | 76 (21.3) | 0.460 |
| Jugular venous pressure, n (%) | 9 (6.3) | 45 (12.6) | **0.042** |
| Third heart sound, n (%) | 13 (9.2) | 43 (12.0) | 0.360 |
| Hepatomegaly, n (%) | 7 (4.9) | 23 (6.4) | 0.520 |
| Echocardiography | | | |
| LVEF (%) | 29.1 (6.7) | 26.6 (7.2) | **<0.001** |
| LVED diameter (mm) | 59.0 (9.2) | 63.7 (9.6) | **<0.001** |
| LVES diameter (mm) | 49.1 (10.0) | 55.0 (10.3) | **<0.001** |
| Left atrial volume (ml) | 92.1 (63.6) | 98.2 (68.3) | 0.580 |
| ECG | | | |
| LBBB (%) | 9 (6.5) | 38 (10.9) | 0.140 |
| RBBB (%) | 5 (3.6) | 26 (7.5) | 0.120 |
| LVH (%) | 35 (25.4) | 62 (18.0) | 0.069 |
| Laboratory | | | |
| eGFR (ml/min^-1^) | 69.8 (54.1-85.0) | 63.0 (43.6-81.9) | **0.031** |
| Sodium (mEq/L) | 139.0 (138.0-141.0) | 139.0 (137.0-141.0) | 0.890 |
| Potassium (mEq/L) | 4.3 (3.9-4.6) | 4.3 (4.0-4.6) | 0.910 |
| Hemoglobin (g/dL) | 13.4 (11.6-15.0) | 13.3 (11.8-14.6) | 0.240 |
| Medication | | | |
| ACEi/ARB, n (%) | 122 (85.9) | 288 (80.7) | 0.170 |
| Beta-blocker, n (%) | 126 (88.7) | 320 (89.6) | 0.770 |
| MRA, n (%) | 77 (54.2) | 236 (66.1) | **0.013** |
| Loop diuretics, n (%) | 88 (66.2) | 287 (82.2) | **<0.001** |
| Digoxin, n (%) | 17 (12.8) | 86 (24.6) | **0.005** |

ACEi, angiotensin converting enzyme inhibitor; ARB, angiotensin receptor blocker; BMI, body mass index; COPD, chronic obstructive pulmonary disease; ECG: Electrocardiography: eGFR, estimated glomerular filtration rate; HFimpEF, heart failure with improved ejection fraction; HFrEF, heart failure with reduced ejection fraction; LBBB, left bundle branch block; LVED, left ventricular end diastolic; LVEF, left ventricular ejection fraction; LVES, left ventricular end systolic; MRA, mineralocorticoid receptor antagonist; New York Heart Association; RBBB, right bundle branch block; LVH, left ventricular hypertrophy.

Values are means ± standard deviations (SD), medians (IQR, interquartile range [25^th^ to 75^th^ percentile range]) or numbers (percentages).

Supplementary Table S5: Predictors of HFimpEF in patients with HFrEF at baseline in ASIAN-HF.

| Predictors | OR | 95% CI | P value[*](https://www.sciencedirect.com/science/article/pii/S0140673619317921?via%3Dihub#tbl3fn1) |
| --- | --- | --- | --- |
| Sex (Female)^#^ | 1.986 | 1.244 - 3.170 | **0.004** |
| Hypertension | 2.512 | 1.590 - 3.967 | **<0.001** |
| Ischemic heart disease^#^ | 0.508 | 0.308 - 0.838 | **0.008** |
| HF hospitalisation in last year | 0.363 | 0.232 - 0.567 | **<0.001** |
| Systolic blood pressure | 1.020 | 1.009 - 1.031 | **<0.001** |
| Diastolic blood pressure | 1.025 | 1.007 - 1.043 | **0.006** |
| LVEF^#^ | 1.050 | 1.017 - 1.084 | **0.003** |
| LVED diameter^#^ | 0.941 | 0.914 - 0.968 | **<0.001** |
| LVES diameter^#^ | 0.933 | 0.908 - 0.959 | **<0.001** |
| MRA^#^ | 0.548 | 0.350 - 0.856 | **0.008** |
| Loop diuretics | 0.446 | 0.275 - 0.723 | **0.001** |
| Digoxin | 0.401 | 0.217 - 0.741 | **0.004** |

CI, 95% confidence interval; LVED, left ventricular end diastolic; LVEF, left ventricular ejection fraction; LVES, left ventricular end systolic; MRA, mineralocorticoid receptor antagonist; OR, odds ratio.

^🞼^Multivariable model: adjusted for age, sex, ischemic heart disease, systolic blood pressure, diabetes mellitus and eGFR.

**^#^**Predictors presented in the BIOSTAT-CHF cohort.Supplementary Figure S1: Overall study design of BIOSTAT-CHF.

**
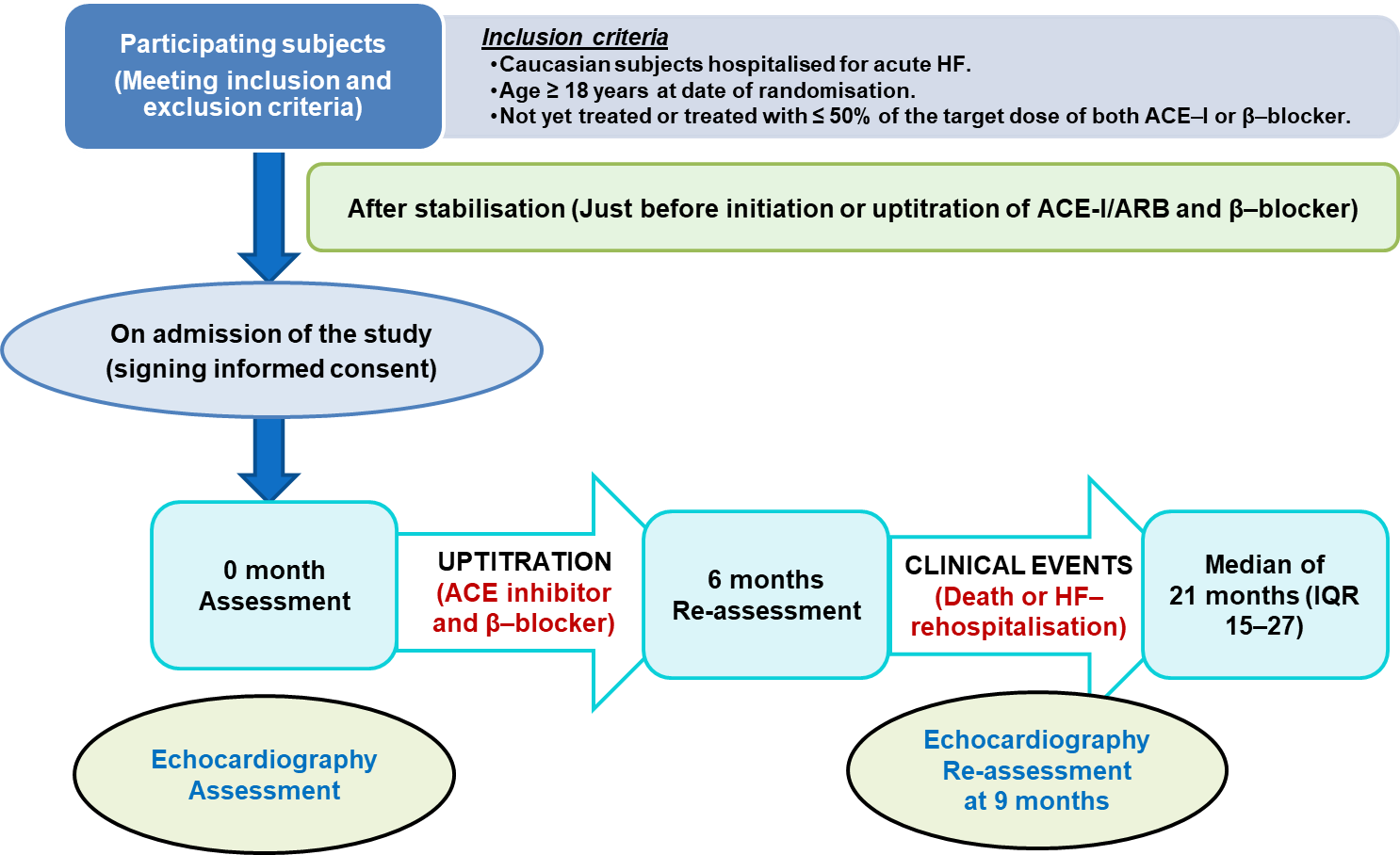
**

**ACEi**, angiotensin converting enzyme inhibitor; **ARB**, angiotensin receptor blocker; **BIOSTAT-CHF**, the BIOlogy Study to TAilored Treatment in Chronic Heart Failure; **HF**, heart failure.

Supplementary Figure S2: Flow diagram of selected patients.

**BIOSTAT-CHF**, the BIOlogy Study to TAilored Treatment in Chronic Heart Failure; **LVEF,** left ventricular ejection fraction; **HFimpEF**, heart failure with improved ejection fraction; **HFrEF**, heart failure with reduced ejection fraction.

**Supplementary Figure S3: Predictive models of HFimpEF in patients who had HFrEF at baseline in BIOSTAT-CHF and ASIAN-HF.**

| **A**  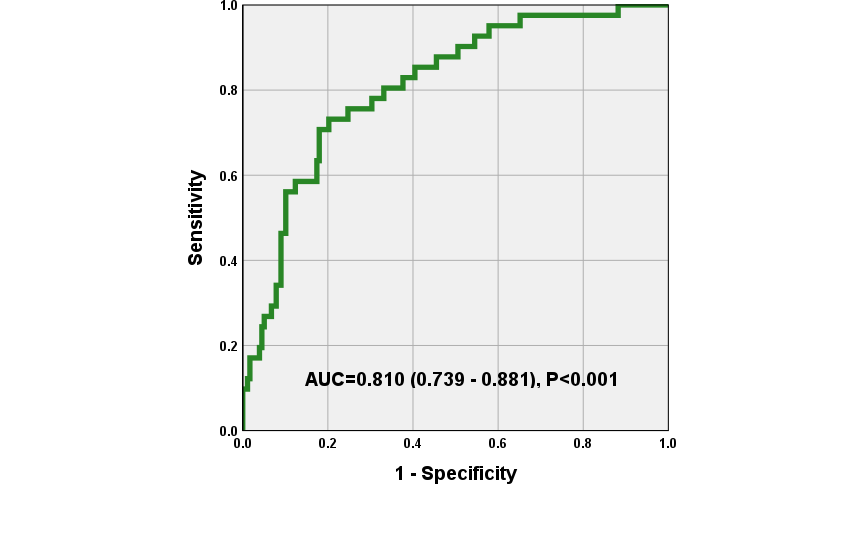 | |
| --- | --- |
| **B**  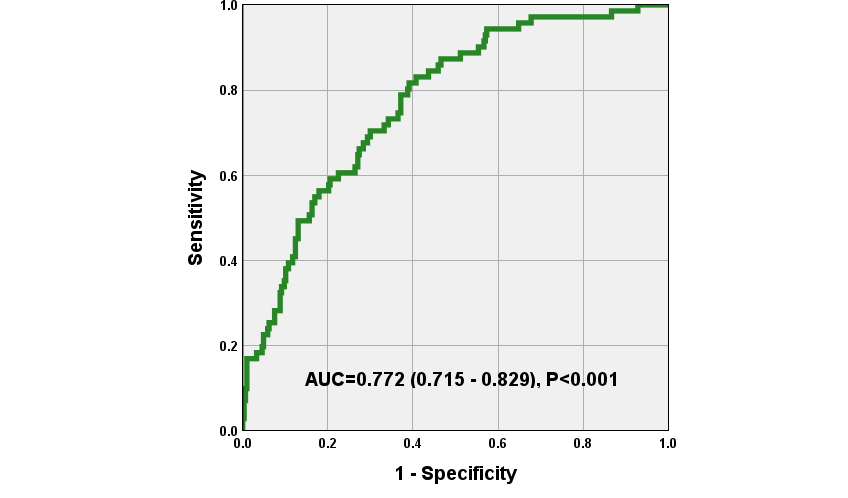 | **C**  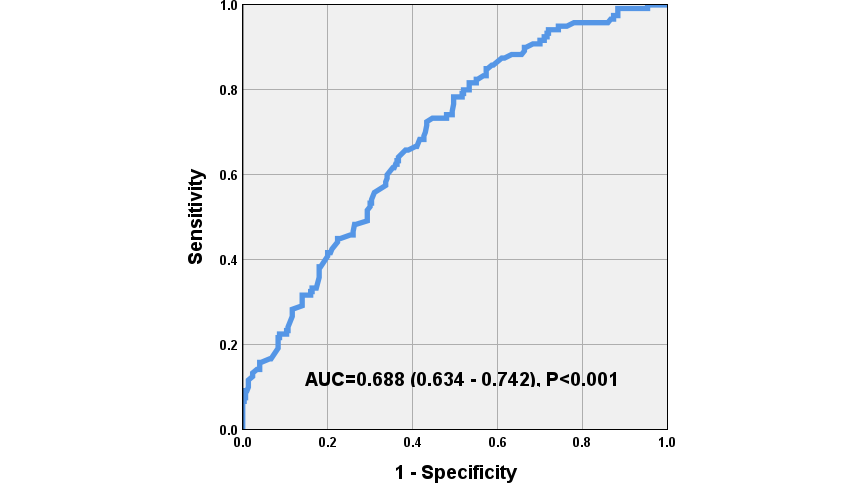 |
| **D**  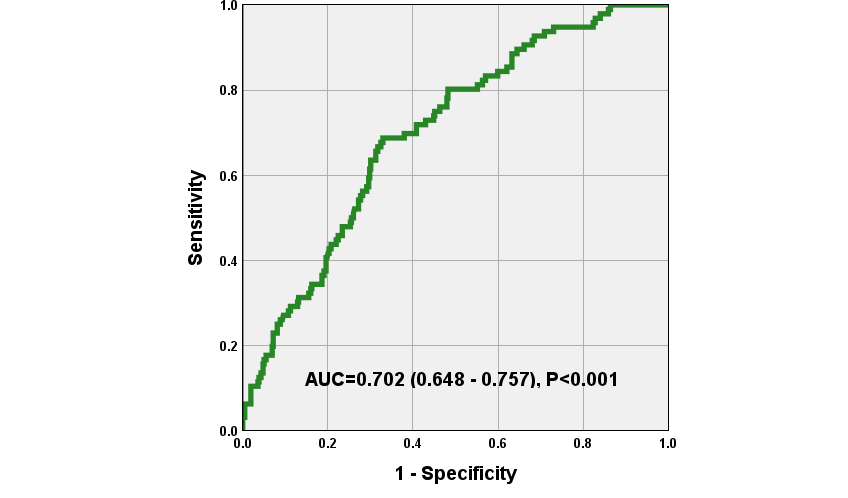 | **E**  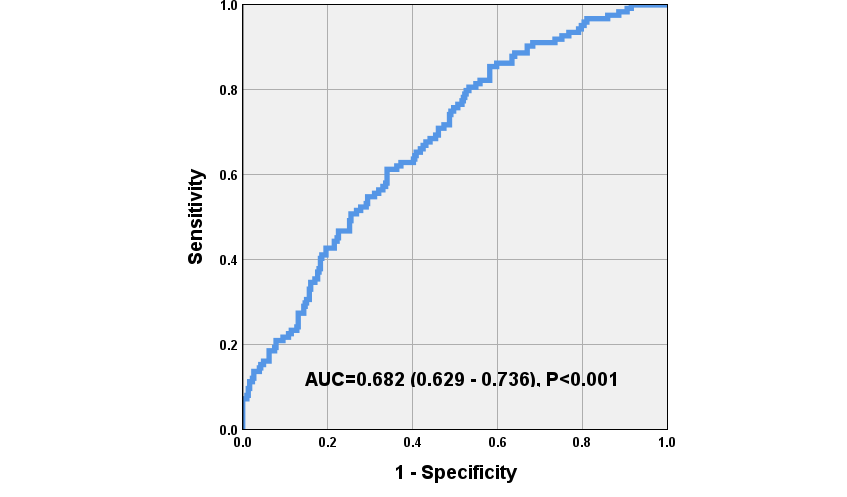 |

**A:** Receiver operating characteristic (ROC) curve of the model in combining ten clinical predictors (including sex, IHD, HR, LVEF, LVED diameter, LVES diameter, LA diameter, LBBB, platelet count, Gamma GT) for prediction of HFimpEF in patients who had HFrEF at baseline in BIOSTAT-CHF.

**B:** Receiver operating characteristic (ROC) curve of the model in combining five clinical predictors (including absence of IHD, smaller LVES diameter, smaller LA diameter, absence of LBBB and higher platelet count) for prediction of HFimpEF in patients who had HFrEF at baseline in BIOSTAT-CHF.

**C:** Receiver operating characteristic (ROC) curve of the model in combining only three clinical predictors (including absence of IHD, smaller LVES diameter and absence of LBBB with missing smaller LA diameter and higher platelet count) for prediction of HFimpEF in patients who had HFrEF at baseline in ASIAN-HF Registry).

**D:** Receiver operating characteristic (ROC) curve of the model in combining five common predictors (including female sex, absence of IHD, higher LVEF, smaller LVED and LVES diameter) for prediction of HFimpEF in patients who had HFrEF at baseline in BIOSTAT-CHF.

**E:** Receiver operating characteristic (ROC) curve of the model in combining five common predictors (including female sex, absence of IHD, higher LVEF, smaller LVED and LVES diameter) for prediction of HFimpEF in patients who had HFrEF at baseline in ASIAN-HF Registry.

**AUC**, area under the receiver operating characteristic curve; **HFimpEF**, heart failure with improved ejection fraction; **HFrEF**, heart failure with reduced ejection fraction.

**Supplementary Figure S4: Clinical outcomes according to HFimpEF (using the 5% LVEF threshold for the HFimpEF classification) and persistent HFrEF in BIOSTAT-CHF.**

| **A** 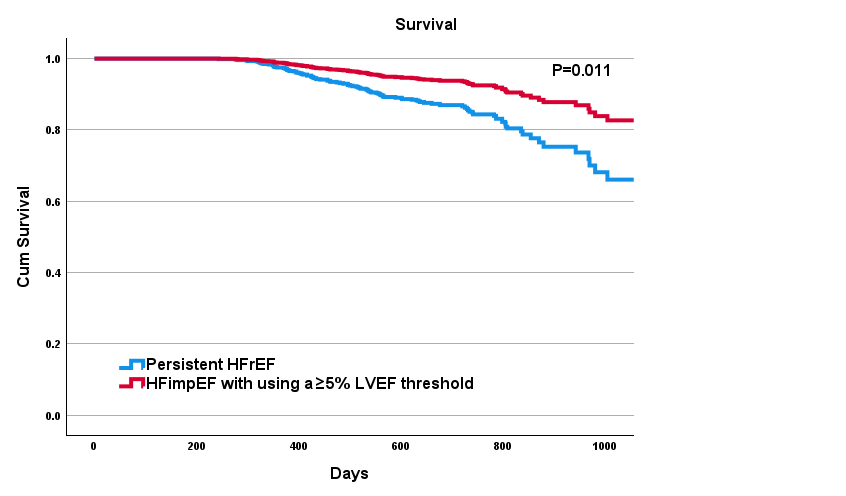 | **B** 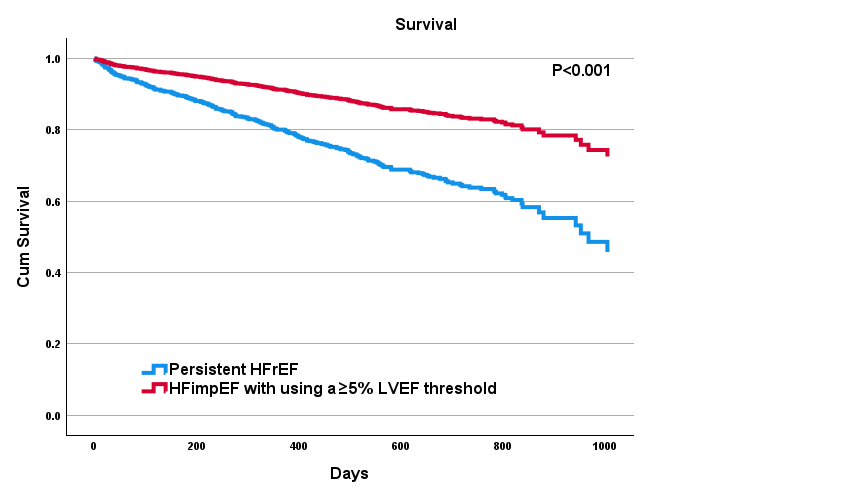 |
| --- | --- |

1. Cox regression survival curves for all-cause mortality according to HFimpEF (using the 5% LVEF threshold for the HFimpEF classification) and persistent HFrEF.
2. Cox regression survival curves for the composite of HF rehospitalisation or all-cause mortality according to HFimpEF (using the 5% LVEF threshold for the HFimpEF classification) and persistent HFrEF.

**HFimpEF**, heart failure with improved ejection fraction; **HFrEF**, heart failure with reduced ejection fraction.
